# Cocaine-and Amphetamine Regulated Transcript (CART) Peptide Is Expressed in Precursor Cells and Somatotropes of the Mouse Pituitary Gland

**DOI:** 10.1371/journal.pone.0160068

**Published:** 2016-09-29

**Authors:** Amanda H. Mortensen, Sally A. Camper

**Affiliations:** Department of Human Genetics, University of Michigan, Ann Arbor, MI, 48109–5618, United States of America; University of Cordoba, SPAIN

## Abstract

Cocaine-and Amphetamine Regulated Transcript (CART) peptide is expressed in the brain, endocrine and neuroendocrine systems and secreted into the serum. It is thought to play a role in regulation of hypothalamic pituitary functions. Here we report a spatial and temporal analysis of *Cart* expression in the pituitaries of adult and developing normal and mutant mice with hypopituitarism. We found that *Prop1* is not necessary for initiation of *Cart* expression in the fetal pituitary at e14.5, but it is required indirectly for maintenance of *Cart* expression in the postnatal anterior pituitary gland. *Pou1f1* deficiency has no effect on *Cart* expression before or after birth. There is no 1:1 correspondence between CART and any particular cell type. In neonates, CART is detected primarily in non-proliferating, POU1F1-positive cells. CART is also found in some cells that express TSH and GH suggesting a correspondence with committed progenitors of the POU1F1 lineage. In summary, we have characterized the normal temporal and cell specific expression of CART in mouse development and demonstrate that postnatal CART expression in the pituitary gland requires PROP1.

## Introduction

CART is expressed in several organs of the neuroendocrine and endocrine system including the pituitary gland, brain, adrenal gland, and the somatostatin producing cells of the pancreatic islets [1–4]. CART is most abundant in the hypothalamus [5]. In rodents, two different splice variants of the *Cart* transcript result in the production of two pro-peptides of different lengths, called proCART 1–89 and proCART 1–102. The proCART peptides contain several cleavage sites that allow post-translational processing by prohormone convertases resulting in two biologically active forms: CART 55–102 and CART 62–102. CART 55–102 is the predominant form in the anterior pituitary gland [5–12]. CART peptides may have a hormonal role as they are found in the pituitary portal blood system and peripheral blood [13], in addition to the anterior and posterior pituitary lobes [1, 14].

CART is thought to function in inhibition of food intake, stimulation of energy expenditure, and regulation of the hypothalamic-pituitary axes [15–19]. In the hypothalamic-pituitary-thyroid (HPT) axis, functional studies in rats and cell lines demonstrate that CART peptide modulates TRH-induced prolactin secretion by influencing the stimulatory affect of TRH [18, 20–22]. There is also evidence that CART regulates the hypothlamic-pituitary-adrenal (HPA) axis at the level of the hypothalamus, where it is expressed together with corticotropin-releasing hormone (CRH) [23]. In vitro studies have shown that CART stimulates the release of CRH from hypothalamic explants [24]. These studies suggest that CART could regulate pituitary function both directly and indirectly.

Several genes have been identified that are required for pituitary development and function in humans and mice [25–27]. Among the best known are *PROP1* and *POU1F1*, which encode two pituitary-specific homeobox transcription factors [28–30]. Mutations in POU1F1 cause Combined Pituitary Hormone Deficiency (CPHD), which is typically characterized by lack of GH, TSH and PRL [30–34], while mutations in PROP1 cause progressive hormone deficiency that can include GH, TSH, PRL, gonadotropins and ACTH [29, 35–39].

Mice with mutations in *Prop1* and *Pou1f1* have been invaluable for revealing the genetic hierarchy of regulatory control and for understanding disease pathophysiology. PROP1 is expressed in Rathke’s pouch, the rudiment of the anterior and intermediate lobes of the rodent pituitary gland, at e10.5 and it wanes by e14.5 [37]. The expression of *Pou1f11* is detectable at e14.5, and expression of *Tshb*, *Gh* and *Prl* are detectable a day later, e15.5 [40, 41]. Ames dwarf mice (*Prop1*^df/df^) have an abnormally shaped Rathke’s pouch and hypoplastic anterior lobe because proliferating progenitor cells are unable to migrate from the niche into the anterior lobe [42–44]. *Prop1*^df/df^ mice also fail to activate *Pou1f1*, and although the Snell dwarf mice (*Pou1f1*^dw/dw^) express *Prop1*, they fail to activate *Tshb*, *Gh* and *Prl* expression [32, 37, 39, 44]. These types of studies, together with lineage tracing experiments, established that *Prop1* is expressed in all pituitary progenitors, it binds the *Pou1f1* regulatory elements and activates its expression, and subsequently, POU1F1 directly activates the hormone genes that define somatotropes, thyrotropes and lactotropes [37, 45].

Identification of target genes is an important step in understanding the molecular mechanisms of transcription factor action. Many downstream targets of POU1F1 have been identified [32, 46, 47], but other than *Pou1f1* and *Hesx1*, few direct targets besides *Prop1* are known [37, 48]. We carried out gene expression profiling with RNA from neonatal pituitaries of normal, *Prop1*^df/df^, and *Pou1f1*^dw/dw^ mice to identify differentially expressed genes that were uniquely changed in *Prop1*^df/df^ relative to the other samples, as these would be candidates for downstream targets of *Prop1*, specifically. The Cocaine- and amphetamine-regulated transcript (CART) was decreased 21 fold in the pituitaries of *Prop1*^*df/df*^ newborns, but no change in *Cart* expression was detected between *Pou1f1*^dw/dw^ and its wild type control [49]. This suggests that CART is a downstream target of *Prop1*, either directly or indirectly.

To determine whether CART is a direct downstream target of *Prop1* and epistatic to *Pou1f1*, we investigated the developmental regulation of CART expression in normal, *Prop1*^df/df^ and *Pou1f1*^dw/dw^ mutant mice. In addition, we investigated the cell specific expression of CART in mouse pituitary. Our results reveal that *Prop1* is not necessary for initiation of *Cart* expression during pituitary embryogenesis, but it is required indirectly for maintenance of *Cart* expression in the postnatal anterior pituitary gland.

## Materials and Methods

### Mice

All mice were housed in a 12-h light, 12-h dark cycle in ventilated cages with unlimited access to tap water and Purina 5020 chow. All procedures were conducted in accordance with the principles and procedures outlined in the National Institutes of Health Guidelines for the Care and Use of Experimental Animals and approved by the University of Michigan Committee for the Use and Care of Animals. Ames dwarf mice (*Prop1*^df/df^) were originally obtained from Dr. A. Bartke (Southern Illinois University, Carbondale, IL) as a non-inbred stock (DF/B), and Snell dwarf mice (*Pou1f1*^dw/dw^) were purchased from The Jackson Laboratory as an inbred stock (DW/J) (Bar Harbor, ME). Both strains have been maintained as colonies at the University of Michigan through heterozygous matings. The morning after conception is designated e0.5 and the day of birth is designated as P1. Mice were euthanized using 10 to 30% carbon dioxide inhalation followed by bilateral pneumothorax.

### PCR genotyping

The genotypes of *Prop1*^df/df^ (p.Ser83Pro) and *Pou1f1*^dw/dw^ (p.Trp251Cys) mice [32, 37] were determined as described [38, 50].

### Tissue Preparation and Histology

Embryos were fixed in 4% formaldehyde in PBS at 4°C for different times depending on the age of the embryo. Fixation was 2 hours for e14.5 and e16.5, overnight for P1 and P8, and 30 minutes for adult mouse pituitaries. The tissue was washed with PBS, dehydrated to 70% ethanol and embedded in a Tissue Tek VIP Paraffin tissue processing machine (Miles Scientific). 6 μm thick sagittal sections were prepared from embryos collected at e14.5 and e16.5, and coronal sections were prepared from postnatal pups and adult pituitaries.

### Immunohistochemistry

Immunostaining for CART protein was done with the anti-CART (55–102) antibody (1:5000, Phoenix Pharmaceuticals). The specificity of the CART (55–102) antibody has been proven with immunohistochemistry experiments were the CART antisera was preabsorbed with the CART peptide [51]. Immunostaining for pituitary hormone markers was performed using anti-TSH, anti-ACTH, anti-LH, anti-FSH, anti-GH, and anti-PRL antibodies, (1:1000, National Hormone and Peptide Program, UCLA Medical Center, Torrance, CA). For double immunostaining of CART and pituitary hormones, the paraffin sections were hydrated and incubated for 20 minutes in an aqueous solution of 3%H_2_O_2_, 50% methanol to block endogenous peroxidases. All slides were placed in normal goat serum block (5% goat serum, 3% BSA, and 0.5% Tween-20 in PBS) for 10 minutes at room temperature. 100 μl of anti-CART antibody was placed on each slide on a wax ring and incubated over night at 4°C. A 100 μl aliquot of biotinylated anti-rabbit IgG secondary antibody (Jackson Immuno Research, West Grove, PA) was incubated on each slide for 30 minutes, and subsequently CART was detected using the tyramide signal amplification (TSA) and Fluorescein Isothiocynate (FITC) kit (according to protocol, Perkin-Elmer, Boston, MA). After washing slides in 1XPBS-T (phosphate buffered saline with 0.01% Tween-20), slides were boiled in 0.01 M citrate buffer to expose epitopes. Specifically, slides and citrate buffer were placed in the microwave for 5 minutes at 40% power. The slides were cooled for 30 minutes in the same citrate buffer [52] After washing the slides in 1X PBS, an Avidin/Biotin block was done for 15 minutes according to protocol (Vector Labs, Burlingame, CA). All hormone antibodies were diluted in this block and were incubated on the slides over night at 4°C, except for GH which was incubated for 1 hour at room temperature. The following secondary antibodies were used: biotinylated anti-guinea pig IgG (1:100,Jackson Immuno Research) for anti-TSHβ, ACTH, LHβ, and FSHβ, biotinylated anti-rabbit IgG (1:100, Jackson Immuno Research) antibody for anti-PRL, and anti-human biotin (1:200, ab97223, Abcam) for anti-GH. The biontinylated secondary antibodies were detected with streptavidin-conjugated Cy3 (Jackson Immuno Research) for 30 minutes at room temperature. Cell nuclei were stained with DAPI (1:200) for 5 minutes, washed with 1XPBS, and mounted with aqueous mounting media.

For double immunostaining with CART and antibodies against the nuclear factors anti-Ki67 (1:200, Novocastra, Newcastel, United Kingdom), anti-TPIT (1:200, from Dr. Jacques Drouin), anti-NR5A1 (1:100, from Ken-ichirou Morohashi), anti-POU1F1 (1:100, from Simon Rhodes), paraffin sections were boiled in 0.01M citrate for 10 minutes, or 15 minutes for anti-PROP1 antibody (1:100, from Aimee Ryan) [45]. These incubations were followed by a 20 minute incubation in an aqueous solution of 3% H_2_O_2_, 50% methanol, and a normal goat serum block (5% goat serum, 3% BSA, and 0.5% Tween-20 in PBS) for 10 minutes at room temperature. 100 μl of CART antibody was placed on each slide within a wax ring and incubated overnight at 4°C and the secondary and tertiary antibodies as described above were used. 100 μl of the nuclear antibodies were added to the slides and incubated over night at 4°C. We used biotinylated anti-rabbit IgG (Jackson Immuno Research) as a secondary antibody for anti-Ki67, anti-NR5A1, anti-TPIT, and anti-POU1F1, and biotinylated anti-guinea pig IgG (Jackson Immuno Resaerch) secondary antibody for anti-PROP1. Nuclear antibodies were detected using either the tyramide signal amplification (TSA) and fluorescein isothiocynate (FITC) kit (according to protocol, Perkin-Elmer, Boston, MA), or streptavidin-conjugated Alexa-fluor 488 (1:200, S11223, Invitrogen). Cell nuclei were stained with DAPI (1:200) for 5 minutes, washed with 1XPBS, and mounted with aqueous mounting media. See Table 1.

**Table 1:** 

Immunohistochemistry experiments									
Experiment	Citric acid boil	CH_3_OH:H_2_O_2_	Block	Primary Antibody	Secondary antibody and detection	Avidin/ Biotin block^8^	5 min Microwave with 10 mM citric acid	Other primary antibody	Other secondary and detection
CART	none	yes	NGS^1^, TNB^2^	1:5000 anti-CART, P.P.^3^	anti-rabbit biotin^4^, TSA FITC^9^	no	no		
CART and POU1F1	10 min	yes	NGS, TNB	1:5000 anti-CART, P.P.	anti-rabbit biotin, TSA TRITC^9^	yes	yes	1:500 anti-POU1F1, gift from S. Rhodes	Anti-rabbit biotin, SA Alexa Flour 488^10^
CART and NR5A1	10 min	yes	NGS, TNB	1:5000 anti-CART, P.P.	anti-rabbit biotin, TSA TRITC	yes	yes	1:1000 anti-NR5A, gift from K. I. Morohashi	Anti-rabbit biotin, SA Alexa Flour 488
ISL1 and TPIT	10 min	yes	NGS, TNB	1:5000 anti-CART, P.P.	anti-rabbit biotin, TSA TRITC	yes	yes	1:200 anti-TPIT, gift from J. Drouin	Anti-rabbit biotin, SA Alexa Flour 488
CART and ACTH	none	yes	NGS, TNB	1:5000 anti-CART, P.P.	anti-rabbit biotin, TSA FITC	yes	no	1:1000, anti-ACTH, NHPP^5^	Anti-guinea pig biotin^4^, SA Cy3^4^
CART and TSHb	none	yes	NGS, TNB	1:5000 anti-CART, P.P.	anti-rabbit biotin, TSA FITC	yes	no	1:1000 anti-TSHb, NHPP	Anti-guinea pig biotin, SA SA Cy3
CART and LHb	none	yes	NGS, TNB	1:5000 anti-CART, P.P	anti-rabbit biotin, TSA FITC	yes	no	1:600 anti-ISL1, DSHB	Anti-guinea pig biotin, SA SA Cy3
CART and GH	none	yes	NGS, TNB	1:5000 anti-CART, P.P	anti-rabbit biotin, TSA FITC	yes	no	1:1000 anti-GH^6^, NHPP	anti-human biotin^7^, SA SA Cy3
CART and FSHb	none	Yes	NGS, TNB	1:5000 anti-CART, P.P	anti-rabbit biotin, TSA FITC	yes	no	1:1000 anti-FSHb, NHPP	Anti-guinea pig biotin, SA SA Cy3
CART and Ki67	10 min	Yes	NGS, TNB	1:5000 anti-CART, P.P	anti-rabbit biotin, TSA TRITC	yes	yes	1:200, anti-Ki67, Novocastra	Anti-rabbit biotin, SA Alexa Flour 488
CART and BrdU	10 min	Yes	TNB, M.O.M.^8^	1:5000 anti-CART, P.P	anti-rabbit biotin, TSA TRITC	yes	yes	1:100, anti-BrDu, AbD Serotec	Anti-rat biotin^4^, SA Alexa Flour 488
PROP1 and CART	15 min	yes^11^	NGS, TNB	1:100 anti-PROP1, gift from A. Ryan	Anti-guinea pig biotin, TSA FITC	yes	yes	1:5000 anti-CART, P.P	anti-rabbit biotin, TSA TRITC

^1^ 5% Normal Goat Serum, 3%BSA in 1XPBS

^2^ Block from Perkin-Elmer TSA FITC kit

^3^ Phoenix Pharmaceuticals

^4^ Jackson Immuno Research

^5^ National Hormone and Pituitary Program

^6^ anti-GH antibody left on for 1 hour

^7^ Abcam

^8^ Vector Labs

^9^ Perkin-Elmer

^10^Invitrogen

^11^ Second CH_3_OH:H_2_O_2_ block done before second primary antibody

Pregnant mice were injected IP with 100mg BrdU per gram of body weight 2 hours prior to collecting embryos. After processing, tissue sections were boiled in 0.01M citrate for 10 minutes, followed by 20 minute incubation in an aqueous solution of 3% H_2_O_2_, 50% methanol. The slides were then incubated for 1 hr in a mouse IgG block. BrdU detection was performed as described by [43] with the anti-BrdU antibody (1:100, AbD Serotec). The secondary biotin anti-rat IgG (1:200, 711-066-152, Jackson Immunoresearch, Westgrove, PA) was used followed by detection with Alexa Flour 488. Microwave treatment, Avidin/Biotin Block, and CART immunostaining were all as described above.

All single and double- immunohistochemistry experiments were done with the appropriate negative controls (S1 Fig).

Cells were counted using ImageJ (1.51a) Software (National Institute of Health).

## Results

### CART is expressed in the developing anterior and posterior pituitary gland

**Fig 1:**
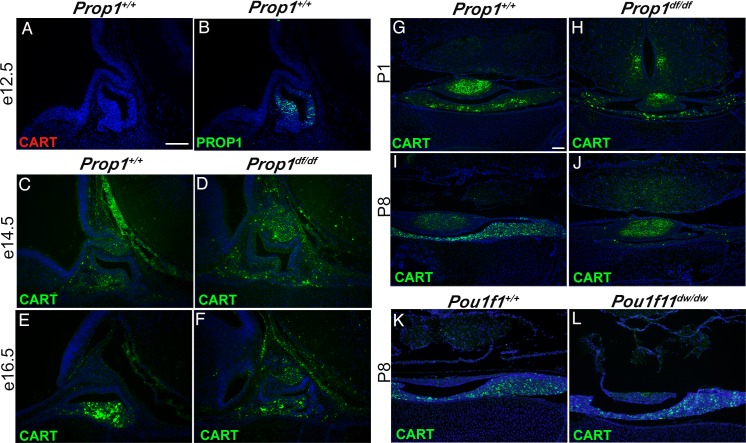
CART is expressed throughout normal pituitary development. CART protein is not detected in e12.5 pituitary gland at e12.5 (A). PROP1 protein is detected in Rathke’s pouch of the e12.5 pituitary gland (B). CART protein was detected in paraffin sections of pituitary glands with a specific antibody (green fluorophor) in *Prop1*^*+/+*^ (C, E, G) and *Prop1*^*df/df*^ (D, F, H) anterior pituitary lobes at e14.5 (C, D), e16.5 (E, F), and P1 (G, H). At P8 CART is expressed abundantly in the anterior pituitary lobe (I), but it is absent in the *Prop1*^*df/df*^ anterior pituitary lobe (J). CART protein is present in the *Pou1f1*^*+/+*^ (normal) (K) and Pou1f1^*dw/dw*^ anterior lobe of the pituitary gland (L). Images A-F were taken at 200X and scale bar is 200 μm. Images G-L were taken at 100X and scale bar is 200 μm.

If *Cart* were a direct target of *Prop1*, then we would expect *Cart* expression to be activated in a spatial and temporal manner coincident or overlapping with *Prop1* expression in normal mice and absent or reduced in *Prop1*^df/df^ mutants. CART was not detected at e12.5 in the pituitary gland when PROP1 is expressed (Fig 1A and 1B). We detected the CART protein at e14.5 in the developing anterior lobe and infundibulum using immunohistochemistry. This pattern persists at e16.5 and the intensity of staining is increased, but there is no striking difference between the control and the *Prop1*^df/df^ mutants at either of these time points (Fig 1C–1F). There are fewer cells that stain for CART in the anterior lobe of *Prop1*^df/df^ at e16.5, but the anterior lobe is hypoplastic at that time. At P1 the CART immunostaining is increasingly prominent in the posterior lobes of both the normal and *Prop1*^df/df^ pituitary glands (Fig 1G and 1H). By P8 CART is detected throughout the anterior and posterior lobe in the control pituitary gland, but it is almost completely absent from the anterior lobe of *Prop1*^df/df^ mutants (Fig 1I and 1J). In contrast, there is no obvious difference between CART expression in control and *Pou1f1*^dw/dw^ mutant pituitary glands at P8 (Fig 1K and 1L). Thus, activation of CART expression is *Prop1-*independent, but *Prop1* is clearly essential for sustaining CART expression in the anterior lobe postnatally. In contrast, *Pou1f1* is not essential for either activation or maintenance of *Cart* expression.

### CART expression predominates in somatotropes of the postnatal pituitary gland

**Fig 2:**
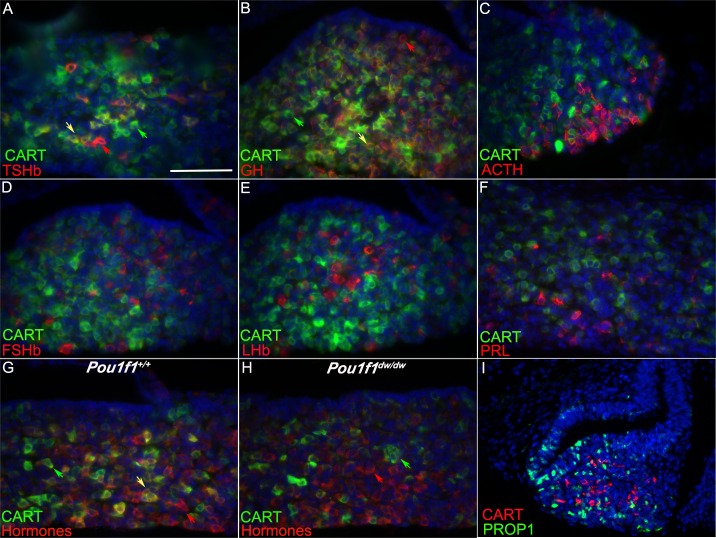
CART expression the pituitary hormones. Co-localization of CART with specific pituitary hormones was determined with a CART-specific antibody (green) and hormone specific antibodies (red) in sections from pituitary glands collected at P8. The hormone specific antibodies were TSHb (A), GH (B), ACTH (C), FSHb (D), LHb (E), and PRL (F). Co-localization is indicated with yellow arrows (A, B). In one CART immunostaining experiment all hormone antibodies were applied together and detected with the same fluorophore (G, H). A few CART positive cells do not express a detectable level of hormone (green arrow) in the *Pou1f1*^*+/+*^ pituitary (G), but almost all CART positive cells do not co-localize with any hormones in the somatotrope deficient *Pou1f1*^dw/dw^ anterior pituitary (H). The CART protein (red) does not co-localize with PROP1 (green) in the developing pituitary gland at e14.5 (I). Images A-I were taken at 400X and scale bar is 100 μm.

We determined the cell specific expression of CART in the pituitary gland at P8 using immunohistochemistry to co-stain for CART and each of the pituitary hormones individually. TSHβwas expressed in 16 to 21% of the CART positive cells in the pituitary gland at P8 (n = 3, Fig 2A, yellow arrow). This is a large portion of the TSHβpopulation as 62 to 79% (n = 3) of the TSHβcells were CART positive. More than half of the CART expressing cells were also positive for GH (57 to 68%, n = 3). (Fig 2B, yellow arrow). There were many GH only cells (31 to 40%, n = 3, red arrow) and CART only cells (green arrow). We did not detect any co-localization of CART with ACTH, LHβ, FSHβ, or PRL at P8 (Fig 2C–2F). The *Pou1f1*^dw/dw^ and *Prop1*^df/df^ mutant pituitaries are nearly devoid of GH, TSHβ, and PRL [41, 44]. It is intriguing that despite identical deficits in *Pou1f1* lineage determination, CART protein expression is normal in *Pou1f1*^dw/dw^ but not detectable in *Prop1*^df/df^ mutants (Fig 1).

To determine whether any of the CART positive cells in the *Pou1f1*^dw/dw^ mutant were producing hormones, we carried co-staining with CART the antibody and an “all hormone” immunostain, by combining antibodies specific for TSHβ, GH, ACTH, LHβ, FSHβ, and PRL and using a common tertiary streptavidin-Cy3 antibody. In the control, 59 to 65% (n = 3) of the CART positive cells co-localized with a hormone producing cell (yellow arrow) (Fig 2G), but all of the CART positive cells were hormone negative in the *Pou1f1*^dw/dw^ pituitaries (Fig 2H). The nature of the CART positive, hormone negative cells is unknown, but the cell morphology resembles that of hormone producing cells.

PROP1 is detectable in progenitors that reside in the marginal zone of the anterior lobe and in scattered cells in the parenchyma of the anterior lobe [53]. Lineage tracing experiments show that all hormone-producing cells of the anterior pituitary gland pass through a *Prop1* expressing progenitor [45]. At e14.5, PROP1 immunostaining is detectable in cells of the most caudal end of the marginal zone and the developing anterior lobe, but expression does not overlap with CART at this time (Fig 2I). This suggests that CART expression may be associated with a progenitor that has initiated commitment to hormone production, but has accumulated low levels of hormone, if any.

### CART co-localizes with multiple cell specific transcription factors, but primarily with POU1F1

**Fig 3:**
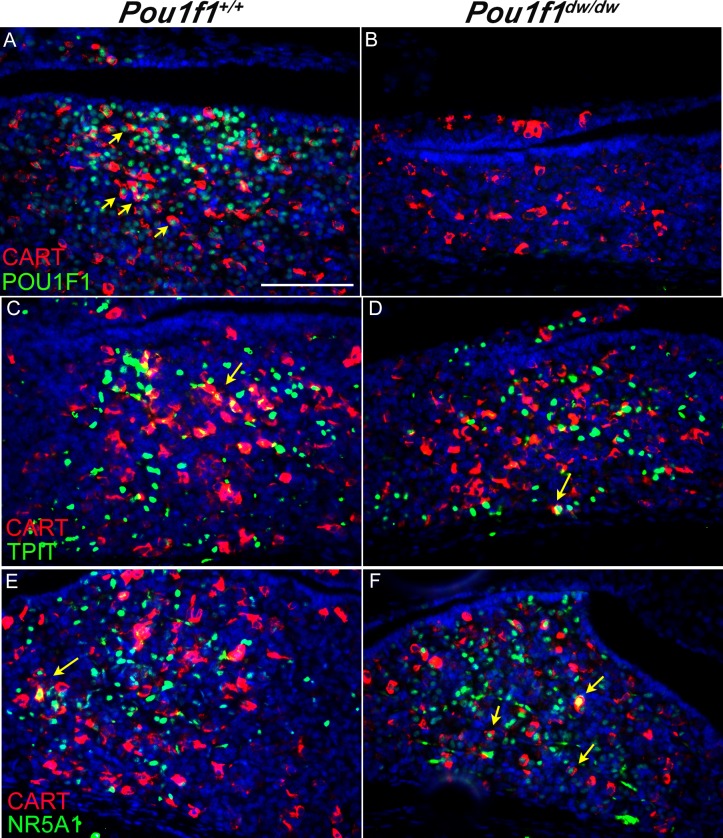
CART is co-expressed with several cell specific transcription factors. Immunostaining with antibodies specific for CART (red, cytoplasmic) and lineage specific transcription factors (green, nuclear) was carried out on paraffin sections of pituitaries collected at P8 from *Pou1f1*^*+/+*^ mice (A, C, E) and *Pou1f1*^dw/dw^ mutants (B, D, F). The lineage specific transcription factors were POU1F1 (A, B), TPIT (C, D), and NR5A1 (E, F). All images were taken at 400X and scale bar is 100 μm.

Some lineage specific transcription factors are detectable prior to the hormone. For example, *Pou1f1* [54] and *Nr5a1 [55, 56]* expression precede the detection of GH and LH respectively [38, 57]. To test whether CART is expressed in committed progenitors, we carried out additional co-immunostaining experiments. At P8 the majority of PROP1 immunostaining is detected in the cytoplasm of cells that have migrated away from the marginal zone, and there is little or no co-localization with CART (data not shown). At P8 CART does co-localize with a large portion of the POU1F1 positive cells in the control pituitary gland (74 to 82%, n = 3, Fig 3A), which supports the idea of a CART expressing progenitor. The CART immunostaining is similar in wild type and *Pou1f1*^dw/dw^ mutant pituitaries, despite the fact that POU1F1 is not functional and not detectable in the mutants (Fig 3B). TPIT (TBX19) is a positive regulator for late POMC cell differentiation and POMC expression, although it is not required for lineage commitment [58, 59]. POMC is processed to produce ACTH in the anterior lobe. We did not detect any co-localization of CART with ACTH at P8 (Fig 2), but we did detect a few TPIT and CART double positive cells in both the control and *Pou1f1*^dw/dw^ pituitary glands (Fig 3C and 3D, yellow arrows). The transcription factor NR5A1 (SF1) is specific to the gonadotropes of the pituitary gland [60]. Although we did not see any co-localization of LHβ or FSHβ with CART, we detected a few CART and NR5A1 positive cells in the control pituitaries (12 to 13% of CART cells, n = 3). We saw slightly more CART and NR5A1 co-localized cells in *Pou1f1*^dw/dw^ mutants (41 to 47% of CART cells, n = 3) compared to the control pituitaries (Fig 3E and 3F, yellow arrow), which may be due to the increased number of cells committed to the gonadotrope fate in these mutants [61]. Taken together these results support the idea that CART is expressed in some committed progenitors that have not yet accumulated hormones. Maintenance of CART expression is indirectly dependent upon *Prop1* and independent of *Pou1f1*, placing it between these two transcription factors in a transcriptional hierarchy.

### CART is expressed primarily in non-proliferating cells of the postnatal pituitary gland

**Fig 4:**
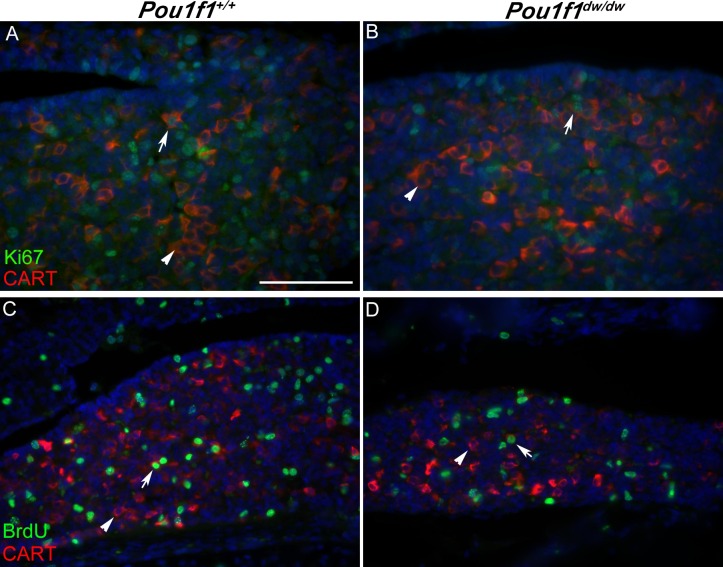
CART is expressed in the proliferating cells of the pituitary gland. Immunohistochemical staining was used to detect CART (red) and the proliferative markers Ki67 (green) or BrdU (green) in sections of pituitaries collect from *Pou1f1*^*+/+*^ and *Pou1f1*^dw/dw^ mice at P8. Co-localization is indicated by white arrows, and CART immunofluorescence in Ki67 or BrdU negative cells is indicated with arrow heads. There are many CART cells in both pituitary genotypes that do not co-localize with any proliferating cells (arrow heads). All images were taken at 400X, and scale bar is 100 μm.

We hypothesized that the CART expressing cells that have no detectable hormone expression could be committed progenitors that are still in a proliferative state. To test this idea we carried out co-immunostaining with antibodies specific for Ki67 and CART at P8. Ki67 marks all active phases of the cell cycle (G_1_, S, G_2_, and M), but it is absent from resting cells (G_0_). Only a small fraction of the CART positive cells were also positive for Ki67 in both the control (8 to 15%, n = 3) and *Pou1f1*^dw/dw^ (12 to 15%, n = 3) pituitary gland (white arrow) (Fig 4A and 4B). We also carried out bromodeoxyuridine (BrdU) labeling at P8 to identify cells in S phase of the cell cycle. Only a small portion of the CART expressing cells also immunostained with BrdU in the control (10–11%, n = 3) and *Pou1f1*^dw/dw^ (9 to 13%, n = 3) (Fig 4C and 4D, white arrow), confirming that the majority of CART expressing cells are non-proliferative. T he few CART expressing cells that are proliferating cells may be among the subset of proliferating, hormone negative, POU1F1 positive cells in the P8 pituitary gland [62].

### CART co-localizes with TSH cells in the adult pituitary

**Fig 5:**
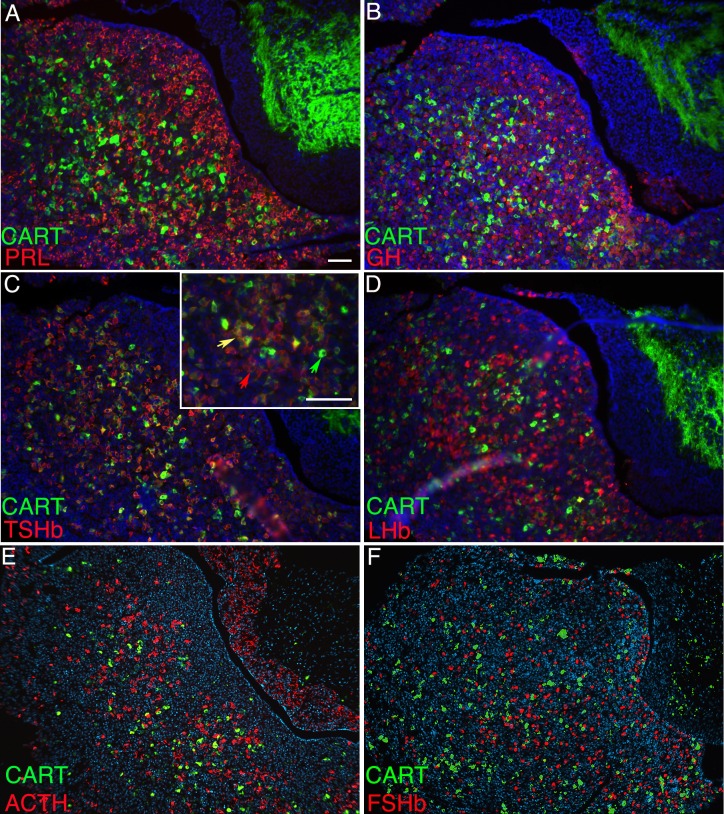
CART co-localizes with thyrotropes in the adult pituitary. Immunohistochemical staining was used to detect co-localization of CART (green) and hormones (red) in sections prepared from adult pituitary glands. The hormone specific antibodies were GH (A), PRL (B), TSHβ (C), LHβ (D), ACTH (E), and FSHβ (F). Representative cells (Inset panel C) exhibited co-localization of CART with TSHβ (yellow arrow), or lacked TSH expression (green arrow), and some TSHβ positive cells lacked CART staining (red arrow). All images were taken at 200X and scale bar is 100 μm. Inset of image C was taken at 400X and scale bar is 100 μm.

We did not detect any co-localization of CART and PRL immunostaining in the pituitary glands of 12 week old adult mice (Fig 5A). In the adult pituitaries there was little to no co-staining of CART and GH (Fig 5B), which contrasts with the abundance co-localization at P8. Many of the CART cells co-localize with TSHβ (yellow arrow) in the adult pituitary (67–76%, n = 3), but CART only (green arrow) and TSHβ only (red arrow) cells are detectable (Fig 5C). We did not observe any co-localization of CART with LHβ, FSHβ, or ACTH in the adult pituitary (Fig 5D–5F).

## Discussion

### *Cart* expression is dependent on *Prop1* after birth, but not embryonically

We identified *Cart* in a screen for genes whose postnatal expression was specifically dependent upon *Prop1* but not *Pou1f1* [49]. Here we report that the initiation and embryonic stages of *Cart* expression are independent of *Prop1*, and the spatial and temporal pattern of *Prop1* and *Cart* expression are inconsistent with *Cart* being a direct target of *Prop1-*mediated transactivation during pituitary embryogenesis. *Prop1* expression is transient in the fetus, diminishing to very low levels by e14.5, but expression is reactivated briefly after birth during a period of rapid pituitary growth and cell proliferation [37, 48, 53]. The postnatal expression of *Cart* is *Prop1* dependent, and we expect that it is indirect because they are not expressed in the same cells embryonically. We expect that the failure to maintain *Cart* expression after birth in *Prop1*^df/df^, but not *Pou1f1*^*dw/dw*^ mutant mice, is due to the arrest in progenitor development at different stages in these two mutants. *Cart* expression may mark cells that have transitioned out of *Prop1* expression and begun commitment to produce hormones, but do not require *Pou1f1* function to maintain *Cart e*xpression. It is possible that initial activation of *Cart* expression involves some of the early acting transcription factors like *Pitx2*, *Lhx3*, or *Lhx4*, [63–65] and that other *Prop1* dependent transcription factors are necessary to maintain its expression postnatally. Candidates for regulating postnatal expression could include *Otx1*, *Notch*, *Math3*, *Zfhx3*, and *Zeb1* [66–70].

### *Cart* expression predominates in the POU1F1 lineage but is not uniquely cell-type specific

*Cart* expression is dynamic and not uniquely cell type specific. *Cart* is first detected at e14.5 in the developing pituitary gland and then persists through adulthood. It does not exhibit exclusive cell specificity at any stage that we examined. We found predominant co-localization in cells expressing *Pou1f1* and GH in neonates, but less co-localization in adult pituitaries. A few cells were detected that express CART and other lineage specific transcription factors like NR5A1 and TPIT, but no co-localization was detected with LH, ACTH or PRL. This developmental shift in co-localization is consistent with the idea that *Cart* marks a *Prop1* dependent progenitor that can give rise to all major hormone producing cell types of the pituitary gland [45]. After *Prop1* activation, mutually antagonistic interactions between POU1F1, NR5A1, GATA2, and probably TPIT (TBX19) may ultimately drive cell specification to different lineages [58, 61]. If this is the case, it could explain why *Cart* expression is detected in a variety of hormone negative committed cells and ultimately becomes more enriched in somatotropes and thyrotropes. It is important to acknowledge that the lack of 1:1 correspondence with CART expression and any particular hormone producing cell type is not unique. The transcriptomes of gonadotropes vary, for example [71].

Given the dynamic *Cart* expression we observed in mice, it is perhaps not surprising that the expression and function reported for CART in the rat anterior pituitary is controversial and differs from the mouse. We did not observe *Cart* expression in mouse lactotropes. However, it was detected in the majority of adult rat lactotropes, expression and secretion of CART 62–102 increased in lactating animals, and CART inhibits TRH-induced prolactin release [20, 21]. *Cart* expression is repressed by thyroid hormone; levels increase in hypothyroid animals and decrease in hyperthyroid animals [20]. Although another group concurs that CART suppresses prolactin release, they detected CART co-localization only with gonadotropes using an antibody specific to CART 55–102, which is the same antibody that we used [22]. Finally, *Cart* transcripts reportedly to co-localize with ACTH cells in the rat, and CART secretion is regulated by CRH and glucocorticoids [72]. There may be species differences in CART expression and function, but the basis for the conflicting co-localization studies in the rat are unclear.

In conclusion, we demonstrate that PROP1 is necessary for maintenance, but not initiation, of CART expression in the pituitary gland. The postnatal maintenance of CART expression by PROP1 is likely indirect. We also show CART co-localization with somatotropes in the mouse pituitary gland, along with non-hormone producing cells that could be progenitors. CART expression could be a useful tool for sorting cells in an intermediate differentiation state.

## Supporting Information

S1 Fig Negative Controls for Co-Immunohistochemistry Experiments. Single antibody immunostaining were conducted as controls in all co-immunohistochemistry experiments. There was no cross-reactivity detected between the CART (55–102) antibody and the other hormone, transcription factor, and proliferation marker antibodies. A, A’, B, B’, C, C’, D, D’, H, H’, I, I’, J, J’, L, and L’ were taken at 400X, scale bar 100 μm. E, E’, F, F’ G, G’ K, K’ were take at 630X, scale bar 100 μm.
(TIF)
