# Supplementary figures and images for: Cocaine-and Amphetamine Regulated Transcript (CART) Peptide Is Expressed in Precursor Cells and Somatotropes of the Mouse Pituitary Gland
Source: PLoS One. 2016 Sep 29;11(9):e0160068. doi: 10.1371/journal.pone.0160068 (PMC5042496; doi:10.1371/journal.pone.0160068)

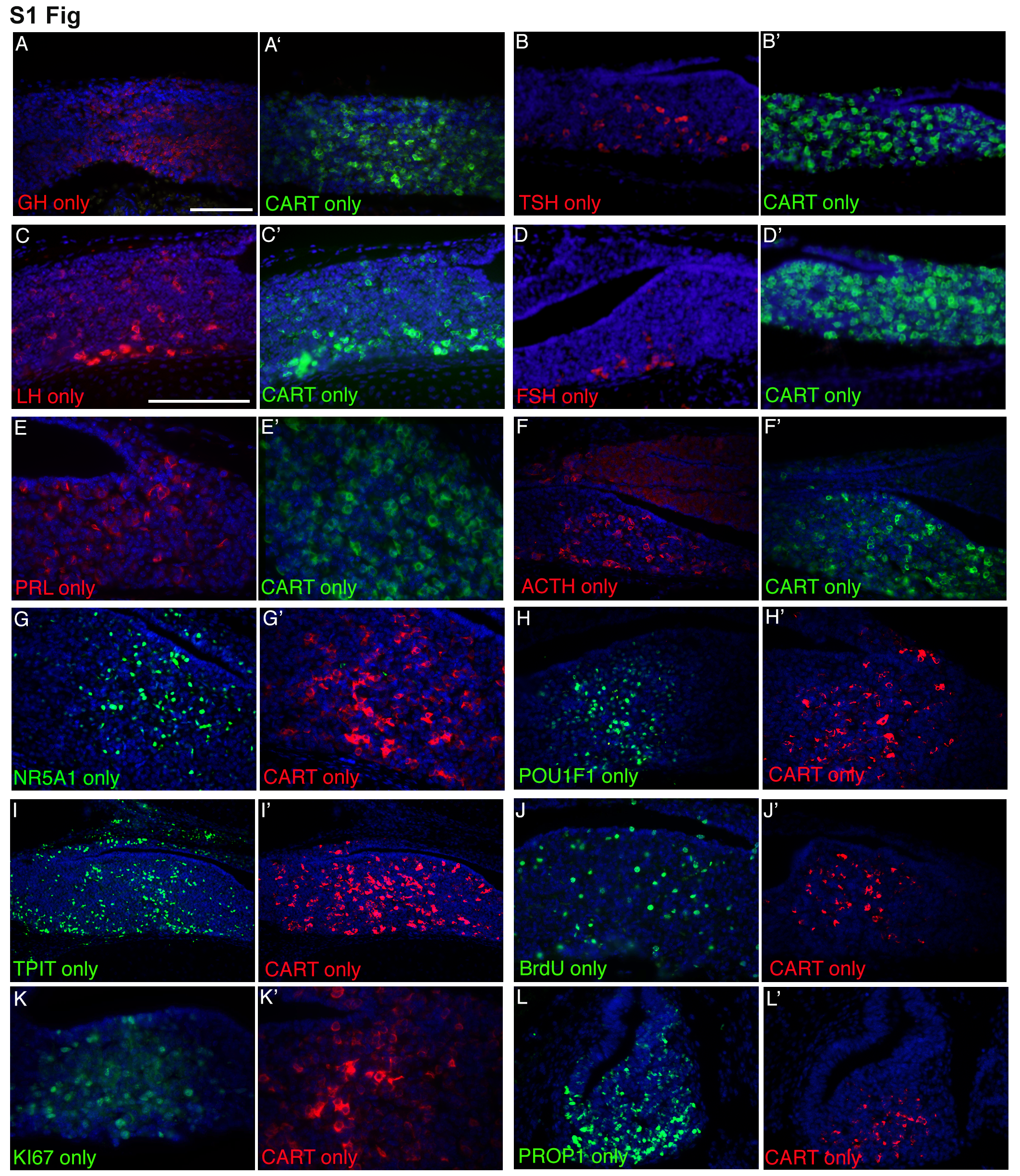

Supplement: S1 Fig — Single antibody immunostaining were conducted as controls in all co-immunohistochemistry experiments. There was no cross-reactivity detected between the CART (55–102) antibody and the other hormone, transcription factor, and proliferation marker antibodies. A, A’, B, B’, C, C’, D, D’, H, H’, I, I’, J, J’, L, and L’ were taken at 400X, scale bar 100 μm. E, E’, F, F’ G, G’ K, K’ were take at 630X, scale bar 100 μm. (TIF) [file pone.0160068.s001.tif]
